# Digestive Characteristics of *Hericium erinaceus* Polysaccharides and Their Positive Effects on Fecal Microbiota of Male and Female Volunteers During *in vitro* Fermentation

**DOI:** 10.3389/fnut.2022.858585

**Published:** 2022-03-31

**Authors:** Baoming Tian, Yan Geng, Tianrui Xu, Xianguo Zou, Rongliang Mao, Xionge Pi, Weicheng Wu, Liangshui Huang, Kai Yang, Xiaoxiong Zeng, Peilong Sun

**Affiliations:** ^1^College of Food Science and Technology, Zhejiang University of Technology, Hangzhou, China; ^2^Changshan Haofeng Agricultural Development Co., Ltd., Quzhou, China; ^3^Zhejiang Academy of Agricultural Sciences, Hangzhou, China; ^4^Research Institute of Changshan Tianle Edible Fungus, Quzhou, China; ^5^College of Food Science and Technology, Nanjing Agricultural University, Nanjing, China

**Keywords:** gut microbiota, short-chain fatty acids, *Hericium erinaceus* polysaccharides, *in vitro* digestion, digestive characteristics

## Abstract

*Hericium erinaceus* polysaccharides (HEPs) have attracted widespread attention in regulating gut microbiota (GM). To investigate digestibility and fermentation of HEPs and their effects on GM composition, three polysaccharide fractions, namely, HEP-30, HEP-50, and HEP-70, were fractionally precipitated with 30%, 50%, and 70% ethanol concentrations (v/v) from hot water-soluble extracts of *Hericium erinaceus*, respectively. Three kinds of prepared HEPs were structurally characterized and simulated gastrointestinal digestion, and their effects on human fecal microbiota fermentations of male and female and short-chain fatty acid (SCFA) production *in vitro* were clarified. Under digestive conditions simulating saliva, stomach, and small intestine, HEPs were not significantly influenced and safely reached the distal intestine. After 24 h of *in vitro* fermentation, the content of SCFAs was significantly enhanced (*p* < 0.05), and the retention rates of total and reducing sugars and pH value were significantly decreased (*p* < 0.05). Thus, HEPs could be utilized by GM, especially HEP-50, and enhanced the relative abundance of SCFA-producing bacteria, e.g., *Bifidobacterium*, *Faecalibacterium*, *Blautia*, *Butyricicoccus*, and *Lactobacillus*. Furthermore, HEPs reduced the relative abundances of opportunistic pathogenic bacteria, e.g., *Escherichia-Shigella*, *Klebsiella*, and *Enterobacter*. This study suggests that gradual ethanol precipitation is available for the preparation of polysaccharides from *Hericium erinaceus*, and the extracted polysaccharide could be developed as functional foods with great development value.

## Introduction

The potential contribution of gut microbiota (GM) to human health has attracted widespread attention over the last decade (1). Studies have shown that some beneficial microbiota in the intestinal tract and its metabolites (e.g., short-chain fatty acids, SCFAs) can regulate intestinal discomfort and promote human health. Bioactive polysaccharides have also been attracted much attention due to their lack of cytotoxicity and various physiological effects (e.g., anti-oxidation, anti-tumor, and immunoregulation) (2). Despite most of the bioactive polysaccharides cannot be utilized by the upper gastrointestinal tract and safely reach into the large intestine, those polysaccharides are decomposed and utilized by the microorganisms (3). Then, the degraded polysaccharides could change the structure of the GM, promote the beneficial metabolites, e.g., SCFA, and lead to improving intestinal health function (4). GM and their fermentative products, such as SCFAs, are considered as metabolic regulators and regulatory agents of complex dietary carbohydrate function and gut health (5). As a major player in the interactions among diet, GM, and the functions of the stomach and intestines, SCFAs have also been reported to contribute to intestinal homeostasis and regulation of energy metabolism. Furthermore, SCFAs regulate intestinal epithelial cell proliferation, maintain epithelial barrier function, modulate immune responses, and help to explain the relationship between GM changes and human metabolic diseases (6). Accordingly, potential strategies or candidates for the use of polysaccharides have been proposed and evaluated by an increasing number of scholars with the aim of regulating the GM and promoting gut health.

*Hericium erinaceus* (*H. erinaceus*) is a large edible fungus cultivated on a large scale, usually taken as a functional food or dietary supplement (7). This fungus is widely used in Asia (used in traditional Chinese medicine). *H. erinaceus* is rich in a variety of active ingredients such as polysaccharides, ketones, sinapine, glycoprotein, and alkaloids (8) and has been demonstrated to possess multiple bioactivities including anti-tumor, gastric ulcer, diabetes, hyperlipidemia, and liver injury (9, 10). The polysaccharides in mushrooms play a prebiotic role, e.g., regulating the GM, metabolizing into SCFAs to increase glucagon-like peptide-1 secretion, and inhibiting gastric emptying function to reduce appetite (11). *H. erinaceus* polysaccharides (HEPs) have been widely reported as the main bioactive compounds, and more than 35 polysaccharides have been isolated and identified (7). Some of them have the activities of anti-oxidation (12), anti-fatigue (13), neuroprotection (14), and immunoregulation (15). HEPs in the fruiting body of *H. erinaceus* could promote the growth of the relative abundance of beneficial GM, e.g., *Lachnospiraceae* and *Akkermansiaceae*, and reduce the abundance of *Rikenellaceae* and *Bacteroids*, which could be used to develop functional food components to accelerate gut health (16). Shao et al. explored how the mycelium of HEPs could adjust the GM of mice with colitis (17). Ren et al. discovered that HEPs alleviated the symptoms of C57BL/6 colitis in mice, which may be attributed to the regulation of oxidative stress, inhibition of inflammation-related signaling pathways, and changes in GM composition (18). *H. erinaceus* extracts (containing polysaccharides) could promote the growth of beneficial gut bacteria and improve the host immunity *in vivo* in the inflammatory bowel disease (IBD) model (19). In a pilot study of population surveys, daily *H. erinaceus* supplementation increased the Alpha diversity within the GM community, upregulated the relative abundance of some SCFA-producing bacteria, and downregulated some pathobionts (20). The *in vitro* intestinal fermentation model is a powerful tool for the study of probiotics and prebiotics on GM, which can simulate different segments of the intestine and sample in real time (21). Simulated *in vitro* digestion and fecal fermentation of polysaccharides can investigate the changes of intestinal microbial environment. It has excellent experimental performance, simple operation, good repeatability, and is not subject to the constraints of human morality. Purification of HEPs is traditionally done by various chromatographic methods including anion chromatography and gel permeation chromatography (GPC) (7). However, these methods usually consume extensive resources and time, which is not ideal in the large-scale purification of polysaccharide. Even though some previous research reported that HEPs could regulate the GM in mice, to date, little information on the digestion and fecal fermentation properties of male and female volunteers is available for different HEP fractions. Therefore, in this study, the *H. erinaceus* was used to grade HEPs by a water extraction-alcohol precipitation process. The changes of total carbohydrate, reducing sugars in simulated digestive system, SCFAs, pH value, and GM communities by high-throughput sequencing *in vitro* fecal fermentation were investigated to explore the prebiotic activity of HEPs.

## Materials and Methods

### Materials and Reagents

The fruiting body of dried *H. erinaceus* was kindly provided by Haofeng Agricultural Development Co., Ltd. (Changshan, Quzhou, China). Ninety-five percent food-grade ethanol was obtained from Changqing Chemical Co., Ltd. (Hangzhou, China). Dialysis tubes (Molecular weight cut-off, 3 kDa) were purchased from Yuanye Biotechnology Co., Ltd. (Shanghai, China). Bile salt, lipase, trypsin, and pepsin were purchased from Macklin Biotechnology Co., Ltd. (Shanghai, China). The basic components of the medium were purchased from Ling Feng Chemical Reagent Co., Ltd. (Shanghai, China). Lastly, other reagents and solvents were purchased from China National Pharmaceutical Industry Co., Ltd. (Shanghai).

Fresh fecal samples were taken from healthy volunteers who did not have history of digestive diseases or any treatment of antibiotics, probiotics, or prebiotics for at least 3 months (five men and five women, all aged between 22 and 25 years old). The volunteers had all been living in Hangzhou, Zhejiang province, and had a traditional Chinese diet. Further, all the volunteers were familiar with the content of the experiment and signed the consent letter in person. The study was approved by the relevant departments of the Ethics Committee of Hangzhou center for disease control and prevention.

### Preparation of the Graded *Hericium erinaceus* Polysaccharides

The HEP preparation refers to the previously reported method with minor modifications (16). Three polysaccharide components were prepared with the same fruiting bodies of *H. erinaceus*. Briefly, the fruiting body of *H. erinaceus* was crushed by high-speed grinders and treated with 95% ethanol for 12 h to remove the pigment, polyphenols, and so on. After filtration and removal of ethanol, it was naturally dried and twice extracted with distilled water (1:15, w/v) at 95°C for 3 h. The combined extracts were centrifuged at 8,000 *g* for 15 min and concentrated by rotary evaporation before 95% ethanol was added gradually in proportion to make the final concentration of 30% ethanol. This was then left to stand at room temperature for 12 h. Next, the precipitate was obtained by centrifugation at 8,000 *g* for 15 min and redissolved in distilled water before protein was removed by the Sevage method (4). Then, the precipitate was dialyzed in a 3 kDa dialysis tube for 3 days, concentrated, and freeze-dried to obtain a crude polysaccharide precipitated with 30% ethanol called HEP-30. After which, a certain amount of 95% ethanol was added to supernatants in proportion to obtain the final ethanol concentration of 50%, and the above experiment was repeated to obtain a crude polysaccharide with 50% ethanol called HEP-50. The preparation of crude polysaccharide, called HEP-70, was obtained by the similar procedure by precipitation with 70% ethanol.

### Determination of Major Components and Structural Characterization of the Graded *Hericium erinaceus* Polysaccharides

The concentrations of total sugar, reducing sugar, uronic acid, and protein were quantified by phenol-sulfuric acid method (16), dinitrosalicylic acid method (DNS) (16), m-hydroxybiphenyl method (16), and coomassie brilliant blue method (22), respectively. The monosaccharide composition of HEPs was analyzed according to the a previously reported method (23), and molecular weight was determined by GPC (23). Compared with the Agilent 1260 Parallax detector, the GPC system (Waters, Milford, MA, United States) consisted of three Waters Ultra-hydrogels, namely, 120, 200 and 500 (7.8 × 300 mm) columns in series. A small amount of samples were completely dissolved in deionized water and then filtrated with a 0.22 μm water phase membrane. The column was eluted with pure water at a flow rate of 1.0 ml/min, and the injection volume of the sample was 40 μl. Fourier Transform Infrared Spectroscopy (FT-IR) analysis of HEPs was scanned on a Nicolet 6700 FT-IR spectrometer (Madison, WI, United States) with potassium bromide (KBr) pellets in a range of 4,000–400 cm^–1^ (24). To compare the microstructure of different HEPs, the apparent morphology of HEPs was observed by a scanning electron microscope (SEM) (Hitachi SU8010, Hitachi Ltd., Japan). In the SEM analysis, a certain amount of dry sample powder was put on the sample plate to spray gold with a vacuum plating apparatus and was observed in a vacuum environment (25).

### Simulation of Saliva Digestion

Saliva was collected from two healthy volunteers (from volunteers who provided fecal samples) before breakfast, centrifuged at 4,500 *g* for 10 min to obtain supernatant, and then mixed with the same volume of HEP solution (8 mg/ml) in a sealed test tube before being kept in a 37°C water bath oscillator. During digestion (0, 5 and 20 min), 1.0 ml of the mixture was removed from the tube and was immediately immersed in boiling water for 5 min to deactivate the enzyme. After the supernatant was obtained by centrifugation, the contents of total and reducing sugar were determined (26).

### Simulation of Gastric Digestion

Based on a previous method (26) 0.05 g CaCl_2_, 0.22 g KCl, 0.62 g NaCl, and 0.12 g NaHCO_3_ were dissolved in 200 ml of distilled water, and pH was adjusted to 3.0 with HCl, i.e., gastric electrolyte solution. Then, 1.0 ml CH3COONa (1 M, pH 5), 37.5 mg gastric lipase, and 35.4 mg pepsin were added into 150 g of gastric electrolyte solution and adjusted pH to 3.0 with HCl, i.e., simulated gastric solution. An equal volume of simulated gastric solution and HEP solution (8 mg/ml) were mixed and kept at 37°C and pH 3.0 in a water bath oscillator. At 0, 2, and 4 h of digestion time, 1.0 ml of the digested sample was immediately immersed in a boiling water bath for 5 min to deactivate the enzyme. After centrifugation, the contents of total sugar and reducing sugar were determined.

### Simulation of Small Intestine Digestion

According to a previous method (4, 26, 27), 0.065 g KCl, 0.033 g CaCl_2_, and 0.54 g NaCl were dissolved in 100 ml distilled water. pH was adjusted to 7.0 with 0.1 M NaOH solution to obtain small intestine medium. Fifty grams of trypsin solution (7%, w/w), 200 g of bile salt (4%, w/w), and 6.5 mg of trypsin were added into the 50 g intestinal medium, in which its pH was adjusted to 7.5 with 0.1 M NaOH solution before being stirred evenly to obtain a simulated intestinal solution. The simulated small intestinal solution and the digested simulated gastric solution were mixed in proportion (3:10). For the simulated small intestinal digestion, the mixtures of 9.0 ml of simulated small intestinal fluid with 30.0 ml of digested simulated gastric solution, 9.0 ml of distilled water with 30.0 ml of digested simulated gastric solution (control group), and 9.0 ml of simulated small intestinal fluid with 30.0 ml of distilled water (control group) were prepared and maintained at 37°C in a water bath oscillator. The digestion samples at 0, 2, and 6 h after simulated intestinal digestion were immediately immersed in a boiling water bath for 5 min to inactivate the enzyme. After centrifugation, the supernatant was taken to measure the concentrations of total and reducing sugar.

### Configuration of Fermentation Medium *in vitro*

One gram of L-cysteine, 2.0 ml of heme, 10 g of tryptone, 2.5 g of yeast extract, 0.9 g of NaCl, 0.0604 g of CaCl_2_⋅6H_2_O, 0.45 g of K_2_HPO_4_, 0.45 g of KH_2_PO_4_, 200 μl of vitamin I solution (biotin 0.05 g/L, cobalamin 0.05 g/L, *p-*amino-benzoic acid 0.15 g/L, folic acid 0.25 g/L, and pyridoxamine 0.75 g/L), and 1.0 ml (1 mg/ml) of resazurin were accurately weighed/measured and dissolved in 1 L of deionized water. Vitamins and L-cysteine were filtered and sterilized, and the rest were boiled and sterilized at 121°C for 15 min. HEP-30, HEP-50, and HEP-70 were used individually as the carbon sources were added into the medium and as an equal weight of fecal diluent were added to the control vessels. Under anaerobic conditions, each bottle was filled 5 ml of fermentation medium and then sterilized. Ten fecal samples from the ten donors were stored separately and grouped by sex in equal amounts (0.8 g) and diluted with pre-reduced anaerobic carbonate-phosphate buffer (feces/buffer 1:10 w/v), followed by filtration through four cheesecloth layers to obtain the fecal slurry. Zero-point-five milliliters of each fecal suspension were inoculated into fermentation media group (HEP-30, HEP-50, HEP-70), respectively. Zero-point-five milliliters (5 parallel tests) of buffer without feces were also treated as the control group. Each fermentation was conducted in triplicate on each of five fecal donors (five men and five women). Finally, all of the fermentation media was maintained at 37°C for 24 h. Next, liquid samples were aliquoted and centrifuged. During the fermentation, 1.0 ml of fermentation broth was taken at 0 and 24 h to determine corresponding parameters. Supernatants were used to determine SCFA production, and the pellets were stored at −80°C for DNA extraction.

### DNA Extraction and High-Throughput Sequencing of Gut Microbiota and Bioinformatic Analysis

The fermentation broth of human feces was centrifuged at 10,000 *g* at 4°C for 5 min to obtain precipitated sample. Total bacterial DNA from fermentation samples of each feces was immediately extracted by the QIAamp DNA Stool Mini Kit (QIAGEN, Valencia, CA, United States) in accordance with the manufacturer’s protocols. DNA concentration and integrity were measured by a NanoDrop 2,000 spectrophotometer (Thermo Fisher Scientific, Waltham, MA, United States) and agarose gel electrophoresis, respectively. The variable regions V3-V4 of the bacterial 16S rRNA genes were performed using the broadly conserved PCR forward primers 338F (5 – ACTCCTACGGGAGGCAGCAG – 3) and reverse primer 806R (5 – GGACTACHVGGGTWTCTAAT – 3) (28). All PCR reactions were carried out in 30 μl reactions with 15 μl of the Phusion^®^High-Fidelity PCR Master Mix (New England Biolabs), 0.2 μM of forward and reverse primers, and about 10 ng template DNA. Thermal cycling consisted of initial denaturation at 98°C for 1 min, followed by 30 cycles of denaturation at 98°C for 10 s, annealing at 50°C for 30 s, elongation at 72°C for 60 s, and, finally, 72°C for 5 min.

After qualified inspection, fluorescence quantification, and library preparation, specific sequencing ligands and PCR amplified products were combined and tested on the Illumina Miseq PE250 high-throughput sequencing platform. The obtained data were further analyzed for corresponding bioinformatic analysis [Mingke Biotechnology (Hangzhou) Co., Ltd., Hangzhou, China] (28). We processed the raw sequence data using QIIME1.9. The function search was used to detect chimerism and remove low-quality sequences. The operational taxonomic unit (OTU, or the sequences with >97% identity) was classified by annotation against the SILVA132 database. We picked representative sequences for each OTU and use the UCLUST to annotate taxonomic information for each representative sequence. In order to compute Alpha Diversity, we rarify the OTU table and calculate three metrics: Chao1 which estimates the species abundance, Observed Species which estimates the amount of unique OTUs found in each sample, and Shannon index. Rarefaction curves were generated based on these three metrics. Graphical representation of the relative abundance of bacterial diversity from phylum to species can be visualized using Krona chart. Principal coordinates analysis (PCoA) of the weighted Unifrac distances was performed using R packages (version 4.1.1) to visualize the compositional differences between the microbial communities of different groups.

### Determination of pH and Air Pressure

The pH value and air pressure of the fermentation samples were quantified with a pH meter (Mettler-Toledo Instruments, China) and a barometer (HT-1895, Dongguan Hongtai Instrument Technology Co., Ltd.), respectively.

### Determination of Short-Chain Fatty Acids

The samples before and after fermentation were centrifuged to obtain the supernatant before being acidified with crotonic acid-metaphosphoric acid mixed solution for 24 h. After acidification, the supernatant was centrifuged and filtered with a 0.22 μm aqueous microporous membrane. The total amount of SCFAs and the content of each component were measured by gas chromatography (GC2010, Shimadzu, Japan) (29).

### Statistical Analysis

Results were shown as mean (3 independent experiments × 3 replication) ± standard deviations (SD). High-throughput sequencing of GM included 5 independent experiments. Origin V.8.5.1 and GraphPad Prism 7 software were used to analyze the data and draw the graph. SPSS V.20.0 was used to conduct analysis of variance (ANOVA) followed by a multiple-comparison test (Duncan test) for the data and difference significance analysis. The level of significance was set at *p* < 0.05.

## Results

### The Component Analysis and Monosaccharide Identification of *Hericium erinaceus* Polysaccharides

The yield and main components of HEPs fractionally precipitated by different concentrations of ethanol are shown in Table 1. With the increase of ethanol concentration, the yield decreased, but there was no significant difference (*p* > 0.05). The content of polysaccharides in HEP-30, HEP-50, and HEP-70 was 46.57% ± 1.70%, 65.01% ± 1.50%, and 58.55% ± 1.40%, respectively. All three groups of HEPs contained a small amount of protein (1.00–1.47%), indicating that the protein was largely removed by the Sevage method.

**TABLE 1:** The main component content of different grades of *Hericium erinaceus* polysaccharides (HEPs).

Group	Yield (%)	Content (%)			
		Crude polysaccharides	Reducing sugar	Protein	Uronic acid
HEP-30	2.03 ± 0.21^a^	46.57 ± 1.70^a^	0.61 ± 0.07^a^	1.00 ± 0.07^a^	1.10 ± 0.20^a^
HEP-50	1.87 ± 0.11^a^	65.01 ± 1.50^c^	0.67 ± 0.08^a^	1.40 ± 0.02^a^	2.40 ± 0.30^b^
HEP-70	1.69 ± 0.15^a^	58.55 ± 1.40^b^	1.63 ± 0.15^a^	1.47 ± 0.05^a^	2.34 ± 0.30^b^

*Data are means ± SD (3 independent experiments × 3 replication). ^a,b,c^ Different lowercase letters in the same column indicate a different significance between treatments (p < 0.05).*

**FIGURE 1:**
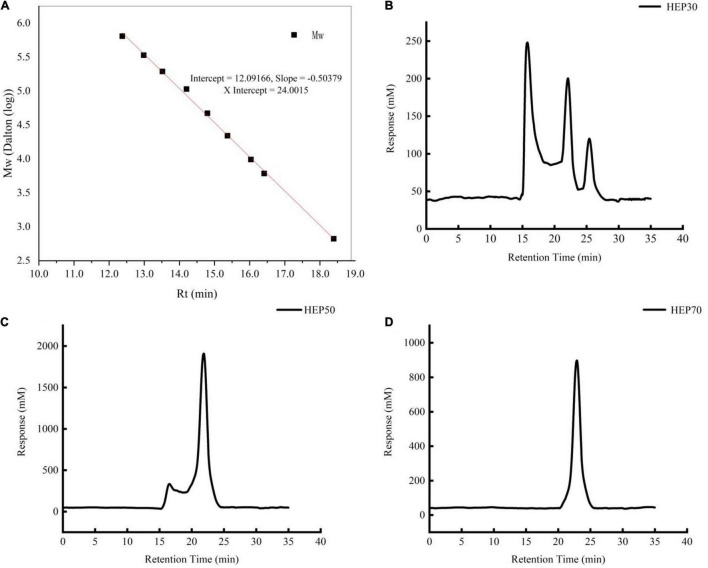
The molecular weight of standard curve. **(A)** *Hericium erinaceus* polysaccharides (HEP)-30 **(B)**, HEP-50 **(C)**, HEP-70 **(D)**.

The molecular weights of HEPs are shown in Figure 1. HEP-30 was mainly composed of three components. The polysaccharide with average molecular weight of 823.07 ± 2.30 kDa (54.36%) had the highest content, followed by 15.019 ± 1.59 kDa. HEP-50 was composed of a main peak and a shoulder peak, with a main molecular weight of 16.723 ± 2.11 kDa (71.68% of the molecular weight segment). Lastly, HEP-70 was relatively pure, with a molecular weight of 4.771 ± 0.21 kDa (95.18% of the total molecular weight). The monosaccharide composition of HEPs is shown in Table 2. All the three grades of HEPs were composed of similar monosaccharide composition in a slightly different molar ratio. The monosaccharide composition of HEP-30 is in the molar ratio of fructose: mannose: glucose: galactose = 0.3: 1.3: 9.8: 0.3; HEP-50 is 1.7: 0.5: 10.6: 10.4; and HEP-70 is 1.2: 1.3: 23.7: 0.3.

**TABLE 2:** Monosaccharide composition of different grades of HEPs.

Group	Monosaccharide (mole ratio)			
	Fucose	Mannose	Glucose	Galactose
HEP-30	0.3	1.3	9.8	0.3
HEP-50	1.7	0.5	10.6	10.4
HEP-70	1.2	1.3	23.7	0.3

### Spectroscopic and Microstructure Analysis of *Hericium erinaceus* Polysaccharides

The FT-IR spectra for HEPs are presented in Supplementary Figure 1. HEP-30, HEP-50, and HEP-70 showed strong absorption peaks generated by the stretching vibration of polysaccharide hydroxyl groups near 3,400 cm^–1^, suggesting that polysaccharides contain a large number of hydroxyl groups in their internal structure and a large number of intermolecular and intramolecular hydrogen bonds (30). The weak absorption peak at 2,900 cm^–1^ is the characteristic absorption caused by the C-H stretching vibration of the methyl in the polysaccharide. The absorption peak at 1,653 cm^–1^ indicates that *C* = O groups or *C* = C groups are existing in the structure (31). The weak absorption peak near 1,400 cm^–1^ is caused by the variable angle vibration of *C* = H, while the asymmetric tensile band at 1653 cm^–1^ and the weaker symmetrical tensile band at 14,00 cm^–1^ are the two bands generated by the carboxylic acid radical groups (32, 33). Absorbance peak at 1,148, 1,096, 1,080, and 1,046 cm^–1^ was classified as pyranose type sugar, and the absorption peak in the range of 1,200–1,000 cm^–1^ was caused by the overlap of the glycoside bond vibration of the sugar ring, C-O-C glycoside bond vibration, and the stretching vibration of the C-OH side group, indicating the presence of pyran glycoside bond in the structure of polysaccharides (34). The absorption peak at 568 cm^–1^ is associated with the sugar cycle (31). The SEM image of HEP is provided in Supplementary Figure 2. The three kinds of polysaccharides are mainly flaky, layered, and smooth with some wrinkles on the surface, while the surface morphologies of the three HEPs presented significant differences in shape and size. In addition, the flaky structure of HEP-30 is a little larger and sparser than the other two polysaccharides, following by HEP-50, which may be attributed to their molecular weight.

### Effects of *Hericium erinaceus* Polysaccharides on Total Sugar and Reducing Sugar Contents

**FIGURE 2:**
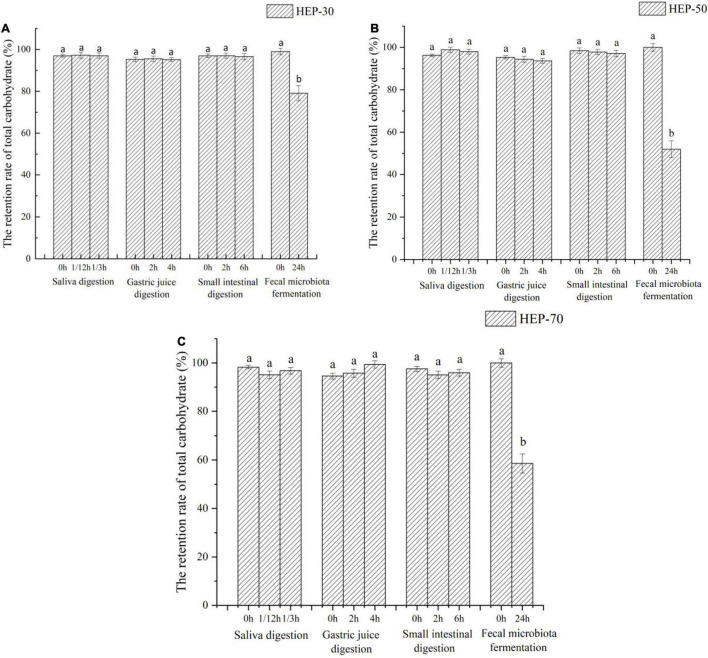
The retention rate of total carbohydrate of HEP-30 **(A)**, HEP-50 **(B)**, and HEP-70 **(C)** during the reaction. The retention rate of total carbohydrate% = (the content of total carbohydrate during the reaction/the content of total carbohydrate before the reaction) × 100%; Data are means ± SD (3 independent experiments × 3 replication). *^a,b^*Different lowercase letters indicate significant differences (*p* < 0.05) between fermentation 0 and 24 h.

**FIGURE 3:**
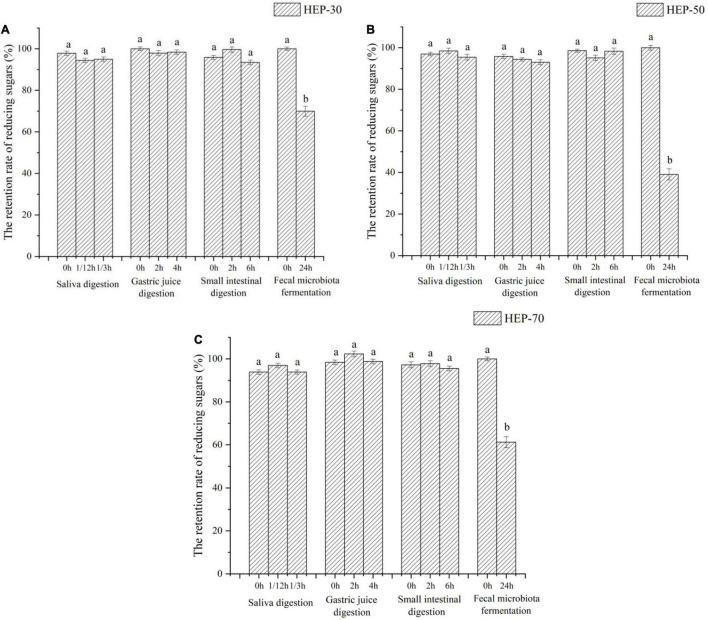
The retention rate of reducing sugars of HEP-30 **(A)**, HEP-50 **(B)**, and HEP-70 **(C)** during the reaction. The retention rate of total carbohydrate = (the content of reducing sugars during the reaction/the content of reducing sugars before the reaction) × 100%; *^a,b^*Data are means ± SD (3 independent experiments × 3 replication). Different lowercase letters indicate significant differences (*p* < 0.05) between fermentation 0 and 24 h.

The changes of total sugar and reducing sugar contents during *in vitro* digestion and fermentation are shown in Figures 2, 3, respectively. According to the data of total sugar retention rate in Figure 2, the retention rates of HEP-30, HEP-50, and HEP-70 in the simulation system of oral cavity, gastric solution, and intestinal solution showed no significant change (*p* > 0.05). However, the total sugar retention rates of the three kinds of polysaccharides were all significantly reduced (*p* < 0.05) after *in vitro* fecal fermentation for 24 h, indicating that HEPs were not easily decomposed by the saliva, gastric juice, and small intestinal juice and could successfully be transported to the distal intestine for microbial fermentation and degradation. After the digestion or fermentation, among the three polysaccharides, HEP-50 had the lowest total sugar retention rate (51.99% ± 4.00%), followed by HEP-70 (58.57% ± 3.99%). The highest retention rate was HEP-30 (76.11% ± 3.60%). It was manifested that HEP-50 might be better degraded and utilized by the GM. According to the retention of reducing sugar in Figure 3, the different grades groups of HEPs showed that there were no significant changes in the simulated digestive solution system (*p* > 0.05). After *in vitro* fermentation for 24 h, there were significant changes (*p* < 0.05) in the retention rates of HEP-30, HEP-50, and HEP-70 (62.53% ± 2.40%, 39.17% ± 2.70% and 60.06% ± 2.60%, respectively), preliminarily concluding that the reducing sugar was absorbed and utilized by GM rather than being digested in the mouth, stomach, or small intestine.

### Effects of *Hericium erinaceus* Polysaccharides *in vitro* Fermentation on Short-Chain Fatty Acids Production, pH and Air Pressure

**FIGURE 4:**
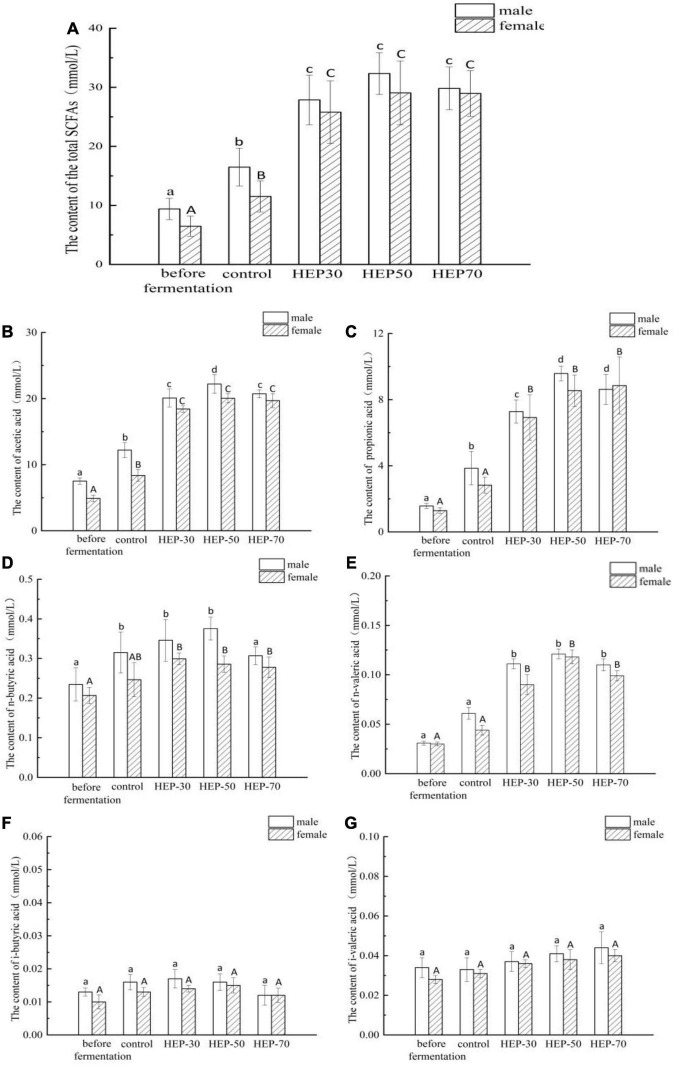
The short-chain fatty acid (SCFA) concentrations of different groups in the feces before or after fermentation. **(A)** total SCFAs; **(B)** acetic acid; **(C)** propionic acid; **(D)** n-butyric acid; **(E)** n-valeric acid; **(F)** i-butyric acid; **(G)** i-valeric acid. Data are means ± SD (3 independent experiments × 3 replication). Different letters (without a common letter) indicated a significant difference between the two experimental groups (*p* < 0.05). ^a,b,c,d^Lowercase and ^A,B^uppercase letters indicate the significance of HEPs on fecal microbiota fermentation of male and female volunteers, respectively.

As shown in Figure 4A, the total SCFAs content in feces of male volunteers was significantly increased compared with that of female volunteers (9.40 ± 1.8 mmol/L *vs.* 6.48 ± 1.7 mmol/L, *p* < 0.05). SCFAs in the fermentation broth of the four groups of media (control group, HEP-30, HEP-50, and HEP-70) after fermentation for 24 h were significantly higher than the samples before fermentation (*p* < 0.05), indicating that SCFAs were metabolized by the GM during fermentation. The content of total SCFAs in the fermentation broth after using HEPs as the carbon source was significantly enhanced compared with the control group, suggesting that HEPs could promote the production of SCFAs by modulating the GM. Hence, the HEP-50 medium had the highest total SCFAs. As shown in Figures 4B–G, after 24 h of fermentation, acetic acid and propionic acid in fecal fermentation broth in male and female volunteers and butyric acid in male volunteers were significantly increased (*p* < 0.05), while the butyric acid in female volunteers, valeric acid, isobutyric acid, and isovaleric acid in female and male volunteers showed no significant changes (*p* > 0.05). Acetic acid and propionic acid are the main metabolites in SCFAs. As per the results, the content of acetic acid is the highest, which is consistent with other relevant reports (17). The HEP treatment significantly increased the contents of acetic acid, propionic acid, and valeric acid compared with the control group after 24 h fermentation (*p* < 0.05).

**FIGURE 5:**
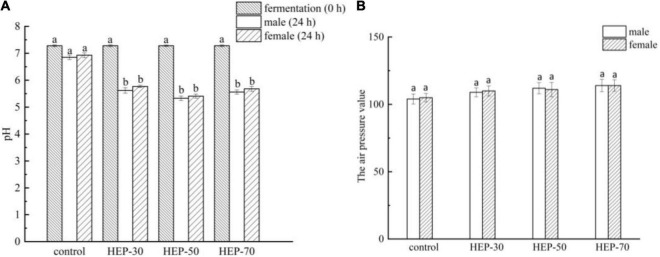
The pH value **(A)** and air pressure **(B)** changed in different groups during *in vitro* fermentation of human feces. Different letters indicated a significant difference between the two groups (*p* < 0.05). Data are means ± SD (3 independent experiments × 3 replication). ^a,b^Different lowercase letters indicate significant differences (*p* < 0.05) between fermentation 0 and 24 h.

As shown in Figure 5A, the initial medium pH was 7.28, and after 24 h anaerobic fermentation, except for the control group, the pH values were significantly reduced in the three HEP-treated groups (*p* < 0.05). The pH difference of the fermentation medium is related to different acids produced by microbes, among them SCFA. The GM can produce SCFAs using polysaccharides, so the difference of SCFA may be associated with the different utilization levels of the three polysaccharides by fecal microbiota. In addition, the pH of HEP-50 group decreased maximally (5.33 ± 0.09 for male and 5.41 ± 0.07 for female). After 24 h of fecal fermentation, the barometer was inserted into the penicillin bottle and the pressure was measured. The results in Figure 5B showed that compared to the control group, the air pressure in the polysaccharides fermentation broth of HEP-30, HEP-50, and HEP-70 groups were not significant changes (*p* > 0.05). It was implied that HEP-30, HEP-50, and HEP-70 groups did not cause abdominal distension, and there was no significant difference between male and female samples (*p* > 0.05).

### Effects of *Hericium erinaceus* Polysaccharides on α and β Diversity of the Fecal Microbiota *in vitro* Fermentation

High-throughput sequencing analysis was performed on 40 samples to explore the effects of HEPs on GM composition. As shown in Supplementary Figure 3A, with the increase of sample sequence number, the slope of the sample curve gradually decreased and finally became flat. This trend reflected that the sequencing depth was reasonable and large enough. Supplementary Figure 3B showed the Shannon-Wiener curve. In the case of this sequencing depth, the sample curves tended to flatten out, indicating that the sequencing data was large enough to evaluate the diversity of microbial species, which explained that the data met the experimental requirements. As shown in Supplementary Figure 3C (Rank abundance curves), there were significant differences in richness and evenness among the communities. The species accumulation curve can be used to judge whether the sample size is sufficient or not. As displayed in Supplementary Figure 3D, the data reflected that the occurrence rate of new OTU (new species) under continuous sampling decreased and the species accumulation curve flattened with the sequencing process, indicating that the sequencing library was large enough to basically cover most bacterial diversity in all samples.

**FIGURE 6:**
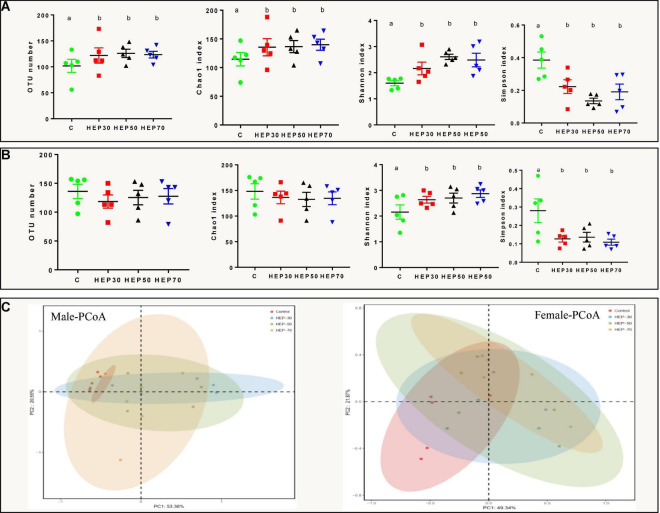
Effects of HEPs on α and β diversity of the fecal microbiota *in vitro* fermentation. α diversity of fecal fermentation broth of male **(A)** and female **(B)** volunteers; **(C)** Principal coordinates analysis (PCoA) of microbiota of fecal fermentation broth *in vitro*. Data are means ± SD (5 independent experiments). ^a,b^Different letters indicated a significant difference between the two groups (*p* < 0.05).

The intestinal community identification of HEP fermentation broth showed that the α diversity and β diversity indexes were significantly different after HEP addition. Figures 6A,B and Supplementary Figure 4 depicted the α diversity of the GM. Compared with the control group, the number of OTUs, Chao1, and Shannon indexes were significantly increased in the fecal fermentation broth of the male volunteers treated with HEPs (*p* < 0.05). In addition, the Simpson index was significantly reduced (*p* < 0.05) (Figure 6A). The fecal fermentation broth of female volunteers treated with HEPs showed a similar trend, with a significant increase in Shannon index (*p* < 0.05) and a significant decrease in Simpson index (*p* < 0.05), but no significant difference was found in the number of OTUs and Chao1 index (Figure 6B). Moreover, as appeared in Supplementary Figure 4, the fecal sample fermentation of the control group for 24 h, the number of OTUs, Chao1, and Shannon indexes of female fecal fermentation broth were significantly (*p* < 0.05) increased compared with those of male fecal fermentation broth, and the Simpson index of female was significantly lower than that in male volunteers (*p* < 0.05). After HEP treatment, the differences of OTU numbers and Chao1 indexes between male and female volunteers disappeared (*p* > 0.05), while the effects of the three polysaccharides on Shannon index and Simpson index between male and female volunteers were different (*p* < 0.05). HEP-30 and HEP-70 did not change the effect on diversity (Shannon index and Simpson index), while HEP-50 eliminated the difference of Shannon index and Simpson index between male and female fecal fermentation broth. As shown in Figure 6C, PCoA analysis showed that HEP had differences in the structure of GM in the feces fermentation broth of male volunteers. The same phenomenon was also observed in the feces of female volunteers, indicating that HEP treatment changed the microbial composition of feces of human volunteers.

### Effects of *Hericium erinaceus* Polysaccharides on the Fecal Microbial Taxonomic Profiles *in vitro* Fermentation

**FIGURE 7:**
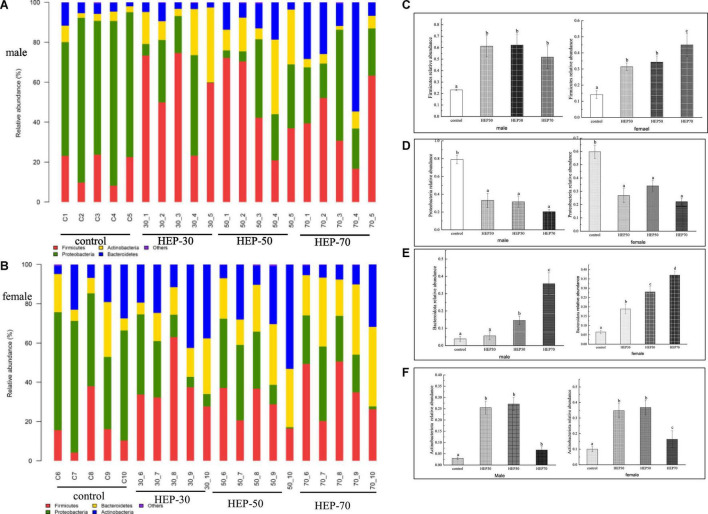
The relative abundance of gut microbiota community at the phylum level *in vitro* fermentation. **(A)**, male; **(B)**, female; **(C)**, Firmicutes; **(D)**, Proteobacteria; **(E)**, Bacteroidetes; **(F)**, Actinobacteria. Data are means ± SD (5 independent experiments). ^a,b,c^Different letters indicated a significant difference between the two groups (*p* < 0.05).

Figures 7A,B and Supplementary Figure 5 are shown the relative abundance at the phylum level of GM (A: male; B: female). As observed from the figures, Firmicutes, Proteobacteria, Bacteroidetes, and Actinobacteria are the main microbial phyla in the fermentation broth of the four media. Actinobacteria was significantly higher in the HEP-30 and HEP-50 groups compared to the control group (*p* < 0.05), and Firmicutes and Bacteroidetes in the fermentation broth of the three groups, except for the HEP-30 group in males, was significantly increased compared with the control group (*p* < 0.05). Meanwhile Proteobacteria was significantly reduced compared with the control group (*P* < 0.05).

**FIGURE 8:**
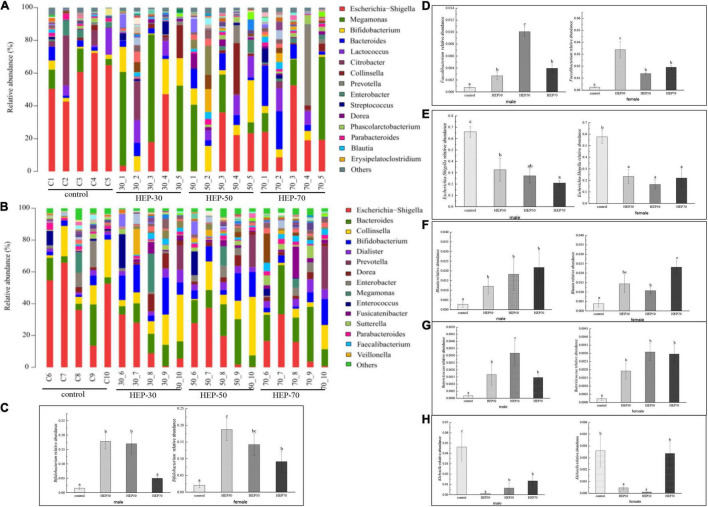
Effects of HEPs on the gut microbiota structure at the genus level. The 15 most abundant at the genus level in male **(A)** and female **(B)**; **(C)**, *Bifidobacterium*; **(D)**, *Faecalibacterium*; **(E)**, *Escherichia-Shigella*; **(F)**, *Blautia*; **(G)**, *Butyricicoccus*; **(H)**, *Klebsiella.* Data are means ± SD (5 independent experiments). ^a,b,c^Different letters (without a common letter) indicated a significant difference between the two groups (*p* < 0.05).

Figure 8, Supplementary Figure 6, and Supplementary Table 1 show the relative abundance of GM at the genus level (A: male; B: female). As seen from the figures, although the GM species of male and female were different, *Bifidobacterium*, *Lactobacillus*, *Faecalibacterium*, and *Prevotella* were increased in both male and female media of the three HEP groups. Moreover, the relative abundance of *Escherichia-Shigella* was decreased compared with the control group. Excluding the HEP-70-treated female volunteers (*p* > 0.05), the overrepresentation of opportunistic pathogenic bacteria, e.g., *Escherichia-Shigella*, *Klebsiella* and, and *Enterobacter* (35), was significantly reduced in the fermentation broth after HEP treatment (*p* < 0.05). Additionally, *Acidaminococcus*, *Bacteroides*, *Dialister* (*p* < 0.05), *Dorea* (*p* < 0.05), *Prevotella 9* (*p* < 0.05), and *Megamonas* (*p* < 0.05) increased in abundance after treatment with HEPs, whereas relative abundances of *Alistipes*, *Collinsella*, *Parabacteroides*, and *Enterobacter* were lower in the HEP-treated group than those in the control group (Supplementary Table 1). It is worth noting that there were gender differences in the influence of HEP on some bacteria, and the influence of HEP fraction on bacteria abundance was also different, but the overall trend of HEP influence on bacteria was the same.

## Discussion

Through the method of hot water extraction and gradual alcohol precipitation, three different grades of HEPs were obtained. All the grades of HEP contained uronic acid, and these data support a previous study (36), but the reason which led to the different contents may be related to the selective type of extraction method, the use of extraction solvent, and the growth environment of the raw materials. The results of molecular weight distribution range of graded HEP was similar to the previous literature (22). Moreover, three graded components were used to study their activity, and Wang et al. also investigated the overall activity of crude polysaccharides (22). The reason for the difference in the molecular weight of the three polysaccharide components should be related to the different concentrations of ethanol precipitation. The lower the concentration of ethanol, the larger molecular weight of polysaccharides that will be precipitated. The monosaccharide composition of HEP, which included fucose, glucose and galactose, was generally consistent with relevant researches (22, 37). However, a small amount of mannose component was also detected in our work, which might be attributed to the different growth environments and preparation methods of *H. erinaceus*. The microstructure of HEP appeared to curl, fold, form irregular aggregates, and can form thin film structures, consistent with other studies in the literature (36). Moreover, the intermolecular crosslinks between HEPs were very tight, which was also characteristic of most plant polysaccharides (38).

The physicochemical and structural properties of polysaccharides change during gastrointestinal digestion. Generally, food digested and absorbed by the host needs to pass through different digestive systems. Furthermore, the retention time of food in the oral cavity is 10 s–2 min, in the stomach is 15 min–3 h, in the small intestine is 2–5 h, and in the large intestine is 12–24 h (39). According to the reference, the simulated digestion time was formulated. In order to explore whether HEPs could safely reach the distal intestine, the simulated digestion ability of HEPs was studied. Some studies have shown that the GM can secrete carbohydrate hydrolase, dramatically decreasing the total sugar content (40). Therefore, the changes in total sugar content are often used as an indicator of the degree of fermentation. Results of the total sugar retention rate were similar to the data of reducing sugar and are also consistent with other relevant reports (4, 27, 41). The reason for the different retention rates of reducing sugar in the three HEP treatment groups may be due to the polysaccharides having produced reducing sugar or oligosaccharides by *in vitro* fermentation. Hence, the different fermentation rates led to different reducing sugar production rates. In addition, fecal microbiota also metabolized reducing sugar. However, the reducing sugar of the fermentation broth treated with HEPs was absorbed and utilized by the GM, which was consistent with the retention result of total sugar content mentioned above, and is also consistent with other related reports (4, 27, 41). The results of HEP in small intestinal digestion were similar to those for saliva and gastric digestion. In a word, all the results suggested that HEPs could pass through the digestive system without being broken down by saliva and simulated gastric and small intestinal digestion and safely reach the large intestine. Thus, it was expected that the GM could metabolize it. However, considering that some oligosaccharides that are usually formed during HEP digestion do not have reductive properties, the use of this standard may underestimate the degree of HEP digestion. This subject is particularly pertinent since the structure and type of glycosidic bonds present in HEP fractions is not known. Therefore, it is difficult to predict the type of oligosaccharide or disaccharide resulting from digestion. It is necessary to further study these contents in the future. The difference of HEP utilization was also related to the structural composition of polysaccharides, e.g., side chain distribution, spatial conformation, degree of polymerization, and monosaccharide composition, or to the physicochemical properties of polysaccharides, e.g., solubility and viscosity (40). Due to its small molecular weight distribution, HEP-70 was easily decomposed, while HEP-50 was not as highly utilized by GM, which may be due to the differences in structure and composition of HEP-70 and the fewer sites of exposed microbial utilization values. Different microbes in the intestinal tract contained different enzymes, regulation, and transport mechanisms, thus specifically breaking down different polysaccharides (42). However, the high retention rate of HEP-30 may be attributed to its relatively large molecular weight, tightly clustered molecular structure, and large molecular volume, which lead to its poor water solubility and difficulty in hydrolysis (43). Current data support that too large or too small molecular weight of HEPs was not conducive to the utilization of GM.

Short-chain fatty acids (SCFAs) are the main metabolites produced by the GM after fermentation of non-degradable carbohydrates which can reduce the gut pH value. Therefore, the intestinal acid environment can suppress the growth of undesirable bacteria and pathogens and affect the enzyme activity of microorganisms. SCFAs can also prevent colorectal cancer degeneration and contribute to maintain gut homeostasis and health (44). Therefore, in this research, the contents of SCFAs were quantified to assess the effect of HEPs on the regulation of the intestinal microenvironment and to further understand the degree of GM decomposition and utilization of HEPs. The present SCFA data also support the results of previous studies *in vivo* (22). These results indicate that the GM can better decompose and utilize HEP-50. Moreover, the results are consistent with the above-mentioned results of carbohydrate utilization. pH is an important indicator that reflects the ability of GM to utilize carbohydrates by fermentation. The pH was consistent with the above SCFA results, indicating that the GM could better utilize HEP-50 components by fermentation.

The structure of GM is crucial for maintaining health. Understanding and exploring the relationship between HEPs and the structure of GM can help to improve human health and prevent some intestinal diseases. The data of α diversity indicate that HEPs can increase the bacterial richness and diversity of male fecal fermentation broth and increase the bacterial diversity of female fecal fermentation broth. This is consistent with population studies which state that daily *H. erinaceus* supplementation increases the Alpha diversity within the GM community (20). According to the previous research data of the population, the richness of the bacterial community is closely linked to obesity, e.g., obese individuals had lower bacterial abundance and gained more weight over time (45). Thus, HEPs showed a beneficial effect in increasing the diversity of GM.

Next, we further analyzed the influence of HEPs on the fecal microbial taxonomic profiles. At the phylum level, HEP treatment increased the relative abundance of Firmicutes, Bacteroidetes, and Actinobacteria to varying degrees, while reducing the relative abundance of Proteobacteria (Figure 7). Bacteroidetes could degrade polysaccharides, increasing their relative abundance (46), possibly indicating that HEP was degraded. In terms of Firmicutes species, many studies have found that the ratio of Firmicutes/Bacteroidetes (F/B) has been related to obesity, but due to the complex relationship of the GM, there is still no clear proportion relationship. Ismail et al. discovered that the F/B value of obese people was higher than that of the normal people (47), while Schwiertz et al. reached the opposite conclusion (48). Therefore, the increase in relative abundance of Firmicutes in this paper was not necessarily related to the obesity phenotype. Moreover, it has been reported that Firmicutes can improve the utilization rate of polysaccharides by participating in glycan degradation through the glycan degradation system (49). Moreover, Proteobacteria is a common pathogenic bacterium that could be harmful to human health (50). Proteobacteria is present at low levels in healthy populations, and its load is considered a potential diagnostic criterion for bacterial dysregulation and disease (50). Many common pathogenic bacteria, e.g., *Escherichia coli*, *Salmonella*, and *Helicobacter pylori*, belong to this phylum (51).

At the genus level, the results of the present study are consistent with *in vivo* studies in animals and human studies that *H. erinaceus* extracts or daily *H. erinaceus* supplementation could promote relative abundances of beneficial GM, e.g., *Bifidobacterium*, *Lactobacillus, Faecalibacterium*, and some SCFA-producing bacteria, and downregulated some pathobionts (19, 20). *Bifidobacterium* is a key symbiotic bacterium that plays a dominant role in human intestines. It can occupy the host soon after birth and is closely related to human health. There have been many studies on the increased abundance of *Bifidobacterium* induced by polysaccharides. In addition, the genome of *Bifidobacterium* contains lots of genes associated with carbohydrate metabolism and shows species-specific adaptability in polysaccharide-rich environments (52). Dietary fiber was conducive to reshaping intestinal microbial ecology, improving ecological imbalance, and enriching the proliferation of SCFA-producing bacteria, e.g., *Prevotella and Bifidobacterium*, thereby increasing the content of SCFAs in feces and systemic circulation. This partially explained the increase of SCFAs in fermentation broth (53). At the same time, *Bifidobacterium* could enhance intestinal barrier function, stimulate the host immune system and probiotic effects on gut function, and weaken the proinflammatory response (54). *Faecalibacterium* was more abundant in the GM of females over males, as butyrate-producing bacteria, and reduced intestinal permeability and inflammation (55). *Faecalibaculum* regulates the metabolic function of the host. Moreover, the present study supported previous research that showed that a high fat and sugar diet reduced the abundance of *Faecalibaculum* and that mannan oligosaccharides increase the abundance of this bacterium (56). Moreover, a 6-month randomized controlled population trial found that low fat diets can increase the α diversity of the GM and the abundance of *Faecalibacterium* (the genus is known to contain butyrate-producing bacteria), whereas the high-fat diet was associated with increased *Bacteroides* and decreased *Faecalibacterium* (57). *Bifidobacterium* and *Lactobacillus* were typical anaerobic probiotics which could degrade macromolecular polysaccharides and have anti-inflammatory and anti-tumorigenic properties (58). Thus, polysaccharides can enhance the proliferation of potential probiotics (*Lactobacillus* and *Bifidobacterium*) in the intestine, reduce the relative abundance of pathogenic bacteria, produce SCFAs, and strengthen immune system of the body (59). In addition, the present data supported that HEP treatment enriched *Blautia* and *Butyricicoccus*, which is consistent with an increase in SCFA levels in feces. *Blautia* is a genus of anaerobic bacteria with probiotic characteristics that widely exists in mammalian feces and intestines and promotes the production of SCFAs and other activities to maintain intestinal homeostasis (51, 60). Moreover, *Butyricicoccus* produce butyrate as energy for gut normal cells, enhance mucosal barrier function, and reduce inflammation levels (51). HEP treatment increased the relative abundance of *Megamonas, Bacteroides*, and *Prevotella* but decreased the abundance of *Alistipes* (Supplementary Table 1). *Megamonas* has not been reported as a dominant genus in intestinal microbiology studies in European and American subjects, but it has been found in studies in China, suggesting that it may be a feature of East Asian populations (61). *Megamonas* genus was found to be positively correlated with the frequency of consumption of beans and serum bilirubin level, suggesting that the genus might be a beneficial microorganism (62). High-fat and low-carbohydrate diet was positively correlated with high risk of cardiac metabolic diseases, and contributed to the growth of *Bacteroides* and *Alistipes*, the reduction of *Faecalibacterium*, the increase of palmitic acid and stearic acid, and the decrease of butyric acid in human feces (63). Previous studies have shown that low-fat diets are associated with an increase in α diversity, *Blautia*, and *Faecalibacterium*, while high-fat diets are linked with an increase in *Alistipes* and *Bacteroides* and a decrease in *Faecalibacterium* (57). In addition, obesity is associated with a reduction in the abundance of intestinal *Bacteroides*, which may be beneficial to health (64). Thus, the abundance of *Bacteroides* should be at a normal level to maintain a healthy intestinal tract. As a dominant genus of bacteria in the human intestine, the *Prevotella spp.* coexist with certain individuals, particularly those with a plant-based diet, and show diversity in the use of a variety of complex carbohydrates (65). More and more people have studied *Prevotella* and regarded it as the beneficial GM (27). Microbial diversity figures reveal that the HEPs can increase the growth of these beneficial bacteria, promoted the growth of probiotics, inhibited the growth of harmful bacteria, improved the GM structure, and adjusted the host health. Physiological impacts of HEPs, both of which pass through the small intestine nearly intact and can be fermented by GM in the large intestine, are similar to each other. They exert a wide range of beneficial effects including anti-inflammation, gut epithelial barrier protection, and immune modulation through both microbiota-dependent and -independent mechanisms (5). Considering these observations, further studies on HEPs will be important for bowel health.

According to our current data analysis, HEPs had basically the same regulation effect on fecal microbiota of male and female volunteers, while the abundance and structure of fecal microbiota are different before fermentation. Therefore, the range of regulation is not completely the same. The effect of HEPs on the sex of volunteers showed a consistent trend. HEPs increased SCFAs and decreased pH value in fecal fermentation broth. The influence of HEP on the Alpha diversity of fecal fermentation broth of different genders was different to some extent, which may be attributed to the difference of Alpha diversity of fecal microbiota before fermentation. For example (Supplementary Figure 4), OTU number, Chao1 index, and Shannon index of female fecal samples before fermentation were significantly higher than those fecal samples of males (*p* < 0.05), while Simpson index was significantly lower than that of males (*p* < 0.05). At the phylum level, the influence trend (increase or decrease) of HEP on the abundance changes of Firmicutes, Proteobacteria, Bacteroidetes, and Actinobacteria was basically the same (Figure 7). At genus level (Figure 8), *Bifidobacterium*, *Faecalibacterium*, *Escherichia-Shigella*, *Blautia*, *Butyricicoccus*, *Klebsiella*, and other sequenced genera (Supplementary Table 1), but there were differences in the amplitude of increase or decrease in the abundance of different genera, which may be attributed to differences in the abundance and structure of the GM before fermentation. Compared with other HEPs, the effect of HEP-50 was relatively effective, but there was no significant difference in multiple indicators. As shown in Table 1, the crude polysaccharide content of HEP-50 was significantly higher than that of the other two HEPs (*p* < 0.05), and there was no significant difference in yield and reducing sugar content (*P* > 0.05), but the content of uronic acid was higher than that of HEP-30 (*p* < 0.05). The composition, structure, and external structure of HEP-50 monosaccharide are also different from those of the other two HEPs, which may be the reason for the difference in effect. Under the digestive conditions simulating saliva, stomach, and small intestine, all three kinds of HEPs were not significantly affected and safely reached the distal intestine. Compared with the other two HEPs, HEP-50 had the lowest retention rate of total sugar and low reducing sugar content in fermentation broth after fecal samples were treated for 24 h, indicating that HEP-50 was more thoroughly utilized and had a better effect on the regulation of fecal microbiota (Figures 2, 3). After HEP-50 fermentation, the total SCFAs and other types of SCFAs (excluding i-valeric acid) were higher than the other two HEPs, indicating that fecal microbes could better decompose and utilize HEP-50. The results were consistent with the aforementioned results of carbohydrate utilization. The effect on pH of fecal fermentation fluid was consistent with SCFA results (Figure 5). In addition, the pH of HEP-50 treatment group was the lowest, but there was no significant difference among groups (*p* > 0.05). For the fecal samples of male volunteers, HEP-50 had a better regulation effect on OTU number, Shannon index, and Simpson index compared to the other two HEPs. Moreover, HEP-50 had a better regulation effect on Chao1 index of fecal samples from female volunteers. However, there was no statistical significance in the regulation of α diversity (*p* > 0.05). At the phylum level, HEP-50 treatment had a better tendency to increase the abundance of Actinobacteria than the other two HEPs in both male and female fecal samples. At the genus level, HEP-50 had significantly better effects on *Faecalibacterium* and *Butyricicoccus* than other HEPs in male fecal samples (*p* < 0.05). In the fermentation broth of female fecal samples treated with HEP-50, *Butyricicoccus* has the highest abundance, while *Klebsiella* and *Escherichia-Shigella* have the lowest abundance.

## Conclusion

The prepared polysaccharide contents of HEPs were 46.57–65.01%, and HEP-50 had the highest total sugar content. HEPs were not digested and utilized in simulated saliva, gastric juice, and small intestinal juice, but it can safely reach the large intestine and be degraded by GM. The effect of HEPs on fecal fermentation broth was basically the same, and there were differences for certain bacteria. After *in vitro* fermentation for 24 h, SCFA concentration was significantly increased, and pH value was decreased in the feces of male and female volunteers treated with HEPs. The phenomenon could be attributed to an increase in SCFA-producing bacteria and beneficial bacteria, such as *Bifidobacterium*, *Faecalibacterium*, *Blautia*, *Butyricicoccus,* and *Lactobacillus*. Furthermore, HEPs reduced the relative abundances of opportunistic pathogenic bacteria, e.g., *Escherichia-Shigella*, *Klebsiella*, and *Enterobacter*. In summary, the above results have shown that HEPs regulate the GM and may be developed as promising prebiotics, which may be conducive to the further development of HEPs in dietary supplement products and health food additives.

## Data Availability Statement

The datasets presented in this study can be found in online repositories. The names of the repository/repositories and accession number(s) can be found below: SRA database, accession number PRJNA806452.

## Ethics Statement

The studies involving human participants were reviewed and approved by the Ethics Committee of the Hangzhou center for diesease control and prevention Approval Code: 202047. The patients/participants provided their written informed consent to participate in this study. Written informed consent was obtained from the individual(s) for the publication of any potentially identifiable images or data included in this article.

## Author Contributions

BT: data curation, writing – original draft, visualization, investigation, and formal analysis. YG and LH: conceptualization. TX: conceptualization, methodology, software, visualization, and investigation. XZ: visualization and methodology. RM: resources. XP: software. WW: validation. KY: project administration, funding acquisition, and writing – review and editing. PS, supervision. All authors contributed to the article and approved the submitted version.

## Conflict of Interest

RM is employed by Changshan Haofeng Agricultural Development Co., Ltd. The remaining authors declare that the research was conducted in the absence of any commercial or financial relationships that could be construed as a potential conflict of interest.

## Publisher’s Note

All claims expressed in this article are solely those of the authors and do not necessarily represent those of their affiliated organizations, or those of the publisher, the editors and the reviewers. Any product that may be evaluated in this article, or claim that may be made by its manufacturer, is not guaranteed or endorsed by the publisher.
