# Supplementary material for: Digestive Characteristics of Hericium erinaceus Polysaccharides and Their Positive Effects on Fecal Microbiota of Male and Female Volunteers During in vitro Fermentation
Source: Front Nutr. 2022 Mar 31;9:858585. doi: 10.3389/fnut.2022.858585 (PMC9008368; doi:10.3389/fnut.2022.858585)
Supplement: Supplementary file 1 [file Data_Sheet_1.docx]

Supplementary Material


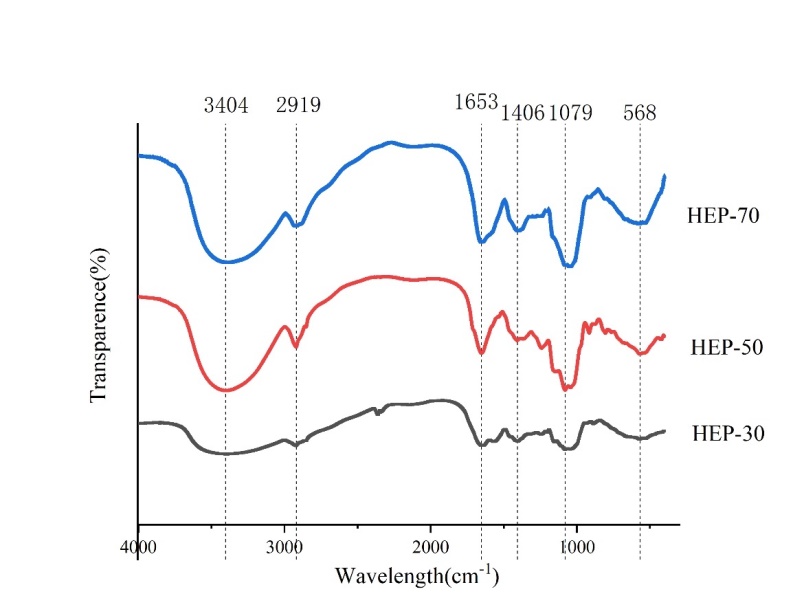


**Supplementary Figure 1.** FT-IR spectrum of different grades of HEPs


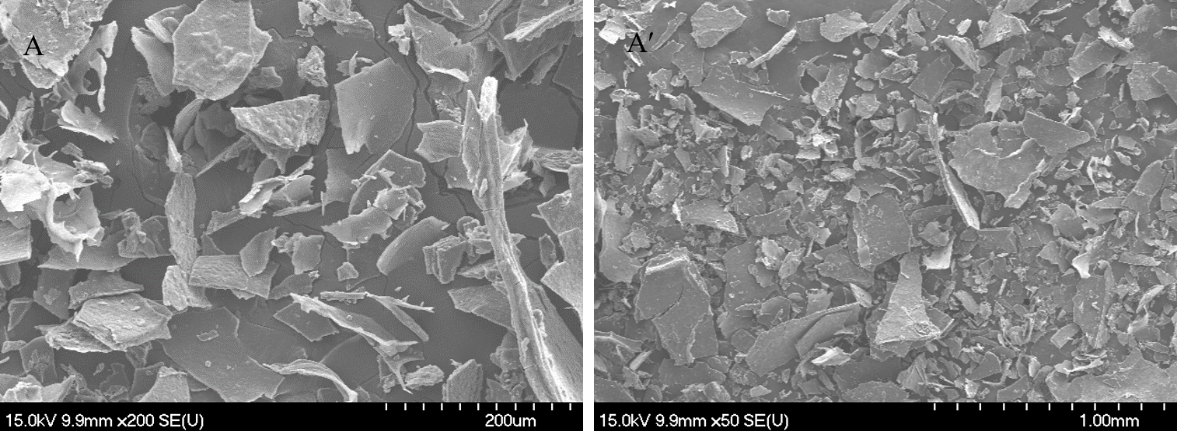


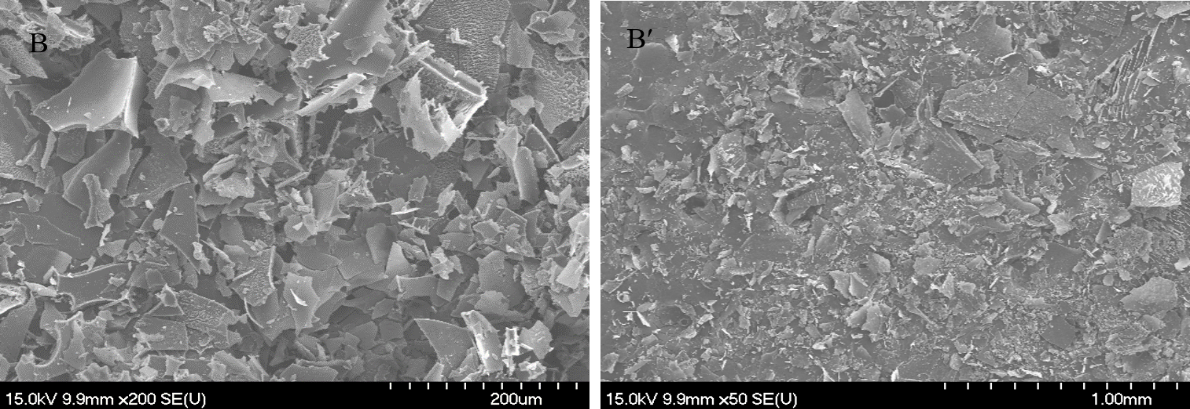


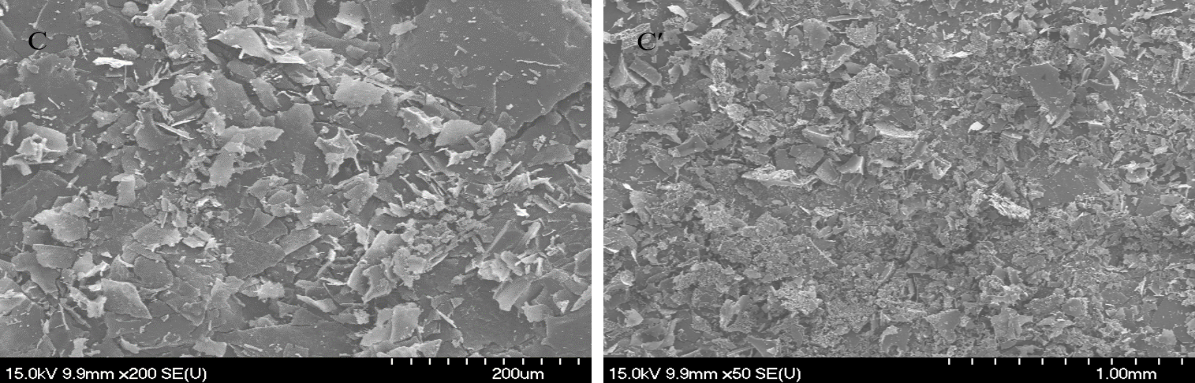


**Supplementary Figure 2.** The SEM pictures of different grades of HEPs. A and A′, B and B′, C and C′ represent the SEM morphology of HEP-30, HEP-50 and HEP-70 at 200 and 50 × magnification, respectively.

**Supplementary Figure 3.** (A) Shannon-Wiener curves of samples; (B) Rarefaction curves; (C) Rank-abundance curves; The abscissa represents the OTU level, and the ordinate represents the relative percentage of sequence number in the OTU level. (D) Species accumulation curves; The abscissa represents the sample size, and the ordinate represents the number of OTU after sampling.

**Supplementary Figure 4.** Effects of HEPs on Alpha diversity in fermentation broth of male and female volunteers in different fecal treatment groups. (A) OTU number; (B) Chao1 index; (C) Shannon index; (D) Simpson index. Data are means ± SD (5 independent experiments). * indicated a significant difference between the two groups (*p* < 0.05).

**Supplementary Figure 5.** The taxonomic heatmap of fecal microbiota *in vitro* fermentation at the phylum level

**Supplementary Figure 6.** The taxonomic heatmap of fecal microbiota *in vitro* fermentation at the genus level

**Supplementary Table 1.** Effects of HEP on the gut microbiota of human fecal fermentation liquid at the genus level

| Genera (%) | | Male | | | | Female | | | |  |
| --- | --- | --- | --- | --- | --- | --- | --- | --- | --- | --- |
|  | control | | HEP30 | HEP50 | HEP70 | control | HEP30 | HEP50 | HEP70 | |
| *Alistipes* | 0.18 ± 0.09a | | 0.03 ±0.02b | 0.07 ± 0.04ab | 0.06 ± 0.03b | 0.38 ± 0.21 | 0.18 ± 0.09 | 0.11 ±0.05 | 0.21 ± 0.10 | |
| *Acidaminococcus* | 0.12 ± 0.10a | | 1.62 ± 0.44b | 1.65 ± 0.48b | 0.43 ± 0.38a | 0.02 ± 0.02a | 0.05 ± 0.04a | 0.10 ± 0.09b | 0.02 ± 0.02a | |
| *Bacteroides* | 3.35 ± 1.31a | | 3.51 ± 1.24a | 7.03 ± 1.68ab | 12.98 ± 4.46b | 10.49 ± 3.91a | 10.92 ± 1.32a | 16.45 ± 3.30ab | 21.68 ± 1.14b | |
| *Lactobacillus* | 0.006±0.003a | | 0.21 ± 0.19b | 0.03 ± 0.01b | 0.01 ± 0.006b | 0.005 ± 0.002a | 0.02 ± 0.004b | 0.013 ± 0.007b | 0.009 ± 0.002b | |
| *Collinsella* | 1.00 ± 0.56a | | 4.82 ± 3.45b | 6.88 ± 0.5.41b | 1.67 ± 0.81a | 11.99±3.73a | 13.38 ± 4.28a | 14.71 ± 5.68a | 5.38 ± 2.33b | |
| *Dialister* | 0.46 ± 0.29a | | 1.12 ± 0.69b | 1.62 ± 0.10b | 1.03 ± 0.54b | 1.76 ± 0.52a | 3.80 ± 1.22b | 3.26 ± 0.78b | 4.66 ± 0.1.19b | |
| *Dorea* | 0.15 ± 0.08a | | 0.93 ± 0.36b | 3.36 ± 1.42b | 1.14 ± 0.34b | 1.25 ± 0.55a | 5.01 ± 1.92b | 3.26 ± 80b | 2.57 ± 0.42b | |
| *Parabacteroides* | 1.90 ± 0.43a | | 0.27 ± 0.08b | 1.48 ± 0.50a | 1.48 ± 0.24a | 2.31 ± 0.49 | 1.09 ± 0.29 | 1.68 ± 0.35 | 2.19 ± 0.35 | |
| *Prevotella 9* | 0.17 ± 0.15a | | 0.50 ± 0.40b | 2.29 ± 1.91c | 10.27 ± 8.74c | 0.03 ± 0.03a | 3.10 ± 2.76b | 4.11 ± 3.68b | 5.44 ± 4.86b | |
| *Megamonas* | 5.99 ± 2.68a | | 35.03 ± 12.93b | 13.92 ± 2.75b | 15.52 ± 8.30b | 2.59 ± 2.31a | 4.85 ± 4.34b | 2.45 ± 2.19a | 1.17 ± 1.05a | |
| *Enterobacter* | 0.69 ± 0.28a | | 0.41 ± 0.22a | 0.60 ± 0.42a | 0.30 ± 0.13b | 5.5 ± 2.83a | 0.86 ± 0.32b | 3.21 ± 1.25b | 2.44 ± 1.20b | |

All values are expressed as the mean ± standard deviation. (n=5 mice/group); differences were assessed by ANOVA followed by multiple-comparison test (Duncan test) for the data and difference significance analysis. Mean value in the same column with ^a,b^different letters (without a common letter) was significantly different (*P* < 0.05).
